# Molecular features of tumor-derived genetic alterations in circulating cell-free DNA in virtue of autopsy analysis

**DOI:** 10.1038/s41598-021-87094-1

**Published:** 2021-04-16

**Authors:** Hayato Koba, Hideharu Kimura, Taro Yoneda, Takashi Sone, Noriyuki Ohkura, Johsuke Hara, Kazuyoshi Hosomichi, Atsushi Tajima, Kazuo Kasahara

**Affiliations:** 1 Cellular Transplantation Biology, Kanazawa University Graduate School of Medical Science, Kanazawa, Japan; 2 Respiratory Medicine, Kanazawa University Hospital, Kanazawa, Japan; 3 Regional Respiratory Symptomatology, Kanazawa University Graduate School of Medical Science, Kanazawa, Japan; 4 Department of Bioinformatics and Genomics, Graduate School of Advanced Preventive Medical Sciences, Kanazawa University, Kanazawa, Japan

**Keywords:** Lung cancer, Tumour biomarkers, Tumour heterogeneity

## Abstract

In cancer patients, circulating cell-free DNA (cfDNA) includes tumor-derived DNA (tDNA). cfDNA has been used clinically for non-invasive gene mutation testing. The aim of this study was to characterize the features of the genetic alterations detected in cfDNA. This study included 6 patients with primary lung cancer who died due to cancer progression. Tumors were biopsied at autopsy. Genetic alteration profiles were obtained using next generation sequencing. The features of the tDNA genetic alterations detected in cfDNA included a higher frequency of being present in multiple tumors (67% truncal mutations, 36% shared mutations, and 4% individual mutations) and a higher variant allele frequency (VAF; 47.6% versus 4.1% for tDNA alterations detected in cfDNA versus not detected in cfDNA, respectively). The data revealed that the tumor-derived genetic alterations most easily detected in cfDNA were truncal mutations with a high VAF. These results showed that essential genetic alterations enriched in cfDNA could help to characterize cancer cells and that genetic testing using cfDNA has advantages in the detection of fundamental regulatory aberrations occurring during tumorigenesis.

## Introduction

Circulating cell-free DNA (cfDNA) is present at very low levels as fragments in the bloodstream. cfDNA includes circulating tumor-derived DNA (ctDNA) in patients with malignancies. Cancer-related genetic alterations can be detected in cfDNA from patients with malignancies^1–6^. Therefore, cfDNA has been clinically examined as a liquid biopsy source in oncogene testing. Liquid biopsies are advantageous because they can be performed safely and repeatedly, and they are less invasive than tumor biopsies. Recently, cfDNA-based mutation tests of the epidermal growth factor receptor (EGFR) gene have been approved as in vitro diagnostic tests in clinical settings worldwide, including in Japan. They are used in treatment decision-making for patients with advanced non-small cell lung cancer (NSCLC). One drawback of cfDNA-based genetic testing is that the detection sensitivity is not always satisfactory^7–9^. The hope is that cfDNA can provide an alternative sample source for genetic testing. Efforts to improve the sensitivity of mutation detection in cfDNA are actively underway. Simultaneously, clarifying the characteristics of genetic alterations detectable in cfDNA will provide useful information for understanding the results of cfDNA genetic testing and effectively using this method in clinical practice.

Although tumor biopsies are essential for pathological diagnosis, and tumor tissue is the first choice for genetic tests conducted in patients with NSCLC, it is unclear whether tissue biopsy specimens are representative of the genetic characteristics of the whole tumor burden of a patient. That is, the biopsy sample is collected from only a small part of the tumor. Samples obtained from transbronchial biopsy, which is one of the most widely used methods of tumor collection in advanced NSCLC, are very limited and represent a small fraction of the tumor lesion. Tumor cells in advanced malignancies exhibit inter- or intratumoral heterogeneity that limits the response to targeted treatments^1^. Low-volume specimens, such as those from transbronchial biopsies, are likely to contain only a small portion of the genetic alterations present; thus, clinicians evaluating these specimens may miss important alterations critical for treatment decision-making because of intra- and intertumoral heterogeneity. In contrast, ctDNA may contain genetic alterations derived from multiple tumor lesions, and ctDNA analyses have the potential to provide an overall picture of all tumor-derived genetic alterations in patients with advanced malignancies, despite tumoral heterogeneity. Although several reports have described analysis of tumor heterogeneity^10,11^, more thorough investigations of tumor genetic alterations detectable in ctDNA are needed.

Next-generation sequencing (NGS) has become a crucial technique for detecting cancer genomic alterations, and it is exploited not only for research purposes but also in the clinic. The major advantage of NGS is its ability to comprehensively analyze disease-related genes to obtain abundant DNA sequence information. Thus, we expect that cfDNA-based genetic testing using NGS should yield promising information by visualizing all of the genetic alterations present in a patient, including those among multiple tumor lesions.

A disadvantage of using cfDNA, compared with tumor tissues, in mutation tests is that the sensitivity of detecting tumor-derived mutations is lower, and the factors influencing detection of tumor-derived genetic alterations in cfDNA are unknown. On the other hand, mutation testing using ctDNA has an advantage over using tumor tissue specimens in that it can potentially detect comprehensive genetic alterations in an individual patient regardless of tumoral heterogeneity. We hypothesized that genetic alterations detected in cfDNA sample analysis could reveal crucial steps in tumorigenesis. To address this hypothesis, in this study, we compared the genetic alteration profiles detected by NGS in tumor tissue autopsy samples with those detected in cfDNA.

## Results

### Patients characteristics

Six patients were enrolled in this study from April, 2011 to September, 2017 and their genetic alteration profiles were analyzed. Three patients were female, and all had smoking histories (20–500 pack-year) except patient 1 (Table 1). All 6 patients had lung adenocarcinoma harboring *EGFR* mutations. Patients received 1 to 11 treatment regimens for NSCLC, and all received EGFR-tyrosine kinase inhibitor (TKI) treatment at least once. The age of the patients at death ranged from 54 to 70 years (Table 2).

**Table 1:** Patient characteristics.

Pt #	Sex	Smoking history (pack-year)	Age at diagnosis-death	Histology at diagnosis	Driver alterations at diagnosis	Treatment regimen for NSCLC
1	F	Never	64–65	Adenocarcinoma	EGFR L858R	Gefitinib, CDDP + PEM, DOC, Erlotinib
2	F	100	50–58	Adenocarcinoma	EGFR Exon 19 deletion	CBDCA + GEM, Gefitinib, PEM + BEV, Erlotinib + PTX, Osimertinib
3	M	500	69–70	Adenocarcinoma	EGFR Exon 19 deletion	Erlotinib, Osimertinib, CBDCA + PTX + BEV, Erlotinib + BEV
4	F	20	51–54	Adenocarcinoma	EGFR L858R	CBDCA + PEM + BEV, DOC, Gefitinib, PEM + BEV, GEM, S-1, Erlotinib, PEM, PEM + BEV, Gefitinib, Afatinib
5	M	70	61–63	Adenocarcinoma	EGFR L858R	Gefitinib, CBDCA + PEM, Erlotinib
6	M	100	68–70	Adenocarcinoma	EGFR Exon 19 deletion	Afatinib

*Pt* patient, *M* male, *F* female, *NSCLC* non-small cell lung cancer, *CDDP* Cisplatin, *PEM* Pemetrexed, *DOC* Docetaxel, *CBDCA* Carboplatin, *GEM* Gemcitabine, *BEV* Bevacizumab, *PTX* Paclitaxel.

**Table 2:** DNA sample characteristics and the gene alterations detected by next-generation sequencing.

Pt #	Tumor samples^a^ and cfDNA (days before death)		Histology	DNA conc. (ng/mL)	Sequence depth^b^	Number of gene alterations	(Both in tumor and cfDNA)^c^	(cfDNA alone)^d^	VAFs of active EGFR mutation (%)
1	Lung	Primary	Squamous carcinoma	1.97	1031	891	9	110	56.9
	Adrenal	Metastasis	Adenocarcinoma	1.43	1110	1235	17	102	82.2
	Soft tissue	Metastasis	Carcinoma	15.4	1431	129	11	108	8.7
	cfDNA	85		5.9	9151	119			27
2	Lung	Primary	Small cell carcinoma	10.4	2380	141	55	119	79.7
	Lung	Metastasis	Adenocarcinoma	5.18	4026	130	78	96	80.1
	Lymph node	Metastasis	Small cell carcinoma	102	3887	155	48	126	19.8
	Liver	Metastasis	Adenocarcinoma	32.8	3708	110	71	103	–
	cfDNA	1		4.4	2487	174			4.7
3	Lung	Primary	Adenocarcinoma	173	3846	97	34	53	40
	Liver	Metastasis	Adenocarcinoma	163	3412	83	29	58	35.2
	Renal	Metastasis	Adenocarcinoma	20.6	3514	140	43	44	–
	cfDNA	0		2.5	8009	87			3.4
4	Lung	Primary	Adenocarcinoma	96.2	2217	201	139	59	55.2
	Lymph node	Metastasis	Adenocarcinoma	84.6	3829	223	135	63	47.3
	cfDNA	21		1.56	8484	198			47.7
5	Lung	Primary	Adenocarcinoma	15.7	1050	68	27	51	–
	Lymph node	Metastasis	Adenocarcinoma	8.12	679	60	21	57	6.8
	cfDNA	68		6.76	1193	78			–
6	Lung	Primary	Adenocarcinoma	171	865	185	95	267	48.5
	Right lung	Metastasis	Adenocarcinoma	205	416	161	94	268	34.3
	Left lung	Metastasis	Adenocarcinoma	161	252	94	48	314	41.7
	Diaphragm	Metastasis	Adenocarcinoma	102	435	93	44	318	42.9
	Liver	Metastasis	Adenocarcinoma	109	363	124	68	294	50.7
	cfDNA	22		22.8	1170	362			12.8

*conc.* concentration, *VAF* variant allele frequency.

^a^Tumor samples were collected from autopsy.

^b^The sequence depth indicates a median number of that in target lesions.

^c^Number of gene alterations both in tumor and cfDNA are listed. It is the number gene of alterations detected in cfDNA among alterations in tumor.

^d^Number of gene alterations in cfDNA alone are listed. It is the number of alterations detected in cfDNA, not in tumor.

### DNA samples used for NGS analysis

DNA from the 6 patients was extracted from 19 total tumor samples (6 primary tumors and 13 metastatic lesions) and 6 cfDNA samples (Table 2). They were used for NGS analyses. DNA in tumor samples (tDNA) was extracted from visible lesions at autopsy. Blood samples were collected at initial diagnosis in four patients (#1, 2, 3, and 6) and before death in all 6 patients. The pre-mortem blood samples were collected at 85, 1, 0, 21, 68, and 22 days before death (patients #1–6, respectively). The primary tumors were adenocarcinoma at initial diagnosis; however, patients #1 and #2 underwent transformation from adenocarcinoma to a different histology. In patient #1, the primary lesion developed into squamous cell carcinoma, whereas one metastatic lesion remained adenocarcinoma. In patient #2, the primary lesion and metastatic lesion in the lymph node developed into small cell carcinoma, whereas the metastatic lesions in the lungs and liver remained adenocarcinoma (Table 2).

### Genetic alteration profiles in tumor tissues and cfDNA using NGS

The DNA samples used in this study were of sufficient concentration and quality to prepare NGS libraries. All NGS libraries derived from tDNA and cfDNA were confirmed to be adequate for sequencing after determining that the molarity and library fragment size (280 bp) were appropriate (Supplementary Fig. 1). The genetic alterations obtained from the sequencing data are summarized in Table 2; the median read depth of the target regions was 1431 (range 252–4026) in the 19 tDNA samples and 5248 (range 1170–9151) in the 6 cfDNA samples. Tumor genotyping assays via NGS were performed using the GENEREAD DNASEQ TARGETED PANELS V2 HUMAN COMPREHENSIVE CANCER PANEL (Qiagen, Valencia, CA, USA), which covers 160 major oncogenes and tumor-suppressor genes (Supplementary Table 1). The mean number of genetic alterations was 218 (range 29–1235) in tDNA and 173 (range 78–362) in cfDNA, and there was no difference in the number of genetic alterations detected by tDNA and cfDNA (Table 2). All genetic alterations profiles from all samples are shown in Supplementary Files 1–31.

### The overlapping genetic alterations between tDNA from different lesions and cfDNA in each patient

**Figure 1:**
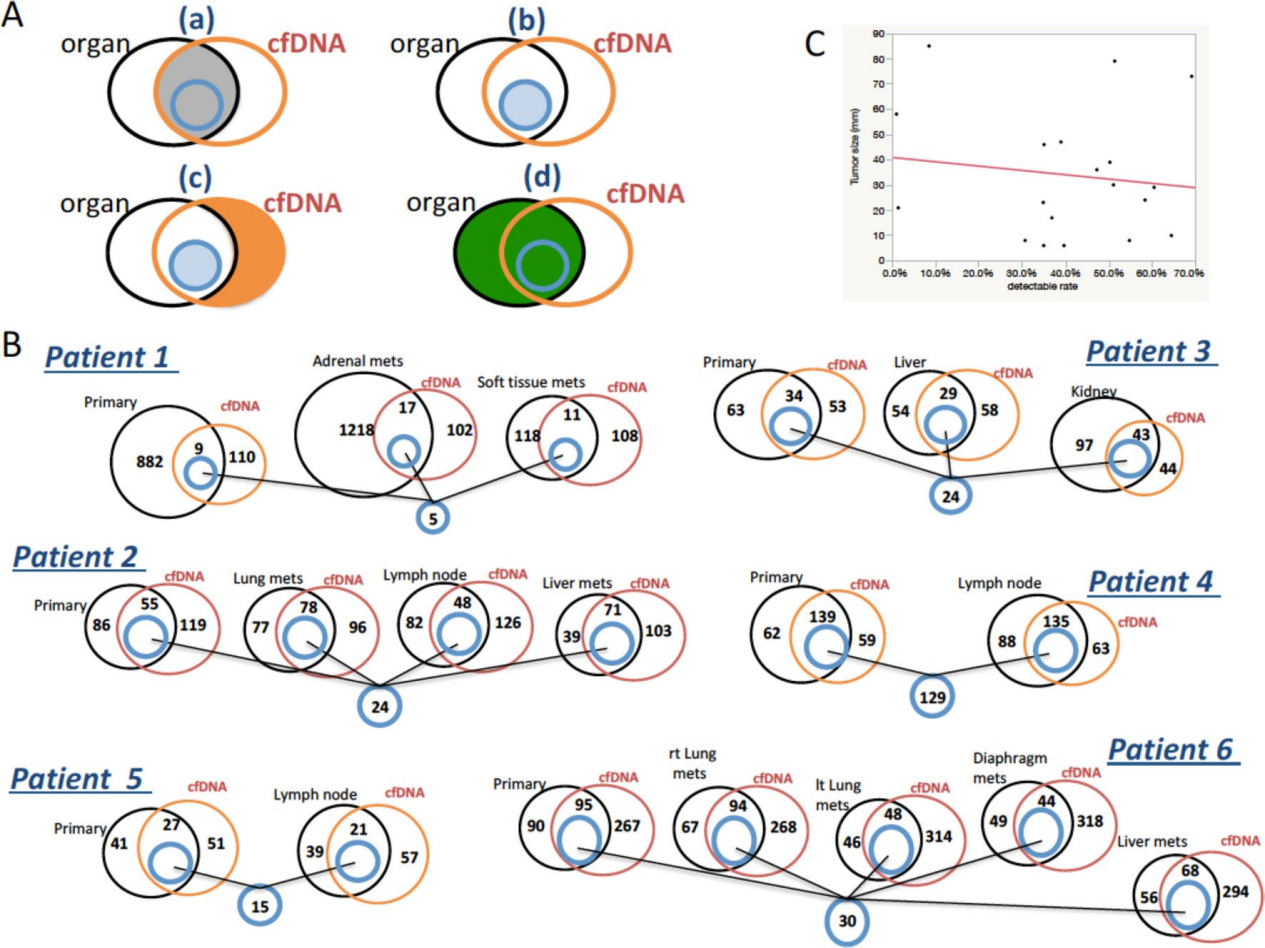
Venn diagrams showing the overlapping genetic alterations detected between tDNA and cfDNA from the same patient. (**A**) The grey area in (a) represents the common alterations detected in both the tDNA and cfDNA samples. The blue area in (b) represents the common alterations detected among each tDNA and cfDNA sample from the same individual patient. The orange area in (c) represents the alterations detected exclusively in the cfDNA samples. The green area in (d) represents all of the alterations detected in the tDNA. (**B**) Overlapping genetic alterations between the tDNA and cfDNA samples of each patient. In patient #3, as an example, of the 97 alterations found in the primary lesion, 34 were also detected in cfDNA, whereas 63 or 53 alterations were identified exclusively in tDNA from the primary lesion or in cfDNA, respectively. Furthermore, there were 24 common alterations among the tDNA samples extracted from the primary lesion and the 2 metastatic lesions and the cfDNA sample of patient #3. (**C**) There was no correlation between the tumor genetic alteration detectability in cfDNA and tumor size. The X axis presents cfDNA mutation detection rate (= grey area/green area in **A**) and the Y axis presents each tumor size. Abbreviations: cfDNA, cell-free DNA; rt., right; lt., left; mets, metastasis.

The common genetic alterations detected in tDNA and cfDNA from the same patient are represented in Venn diagrams in Fig. 1. The overlapping portions of the circles represent the number of alterations with completely matching nucleotide changes between tDNA and cfDNA (gray areas in Fig. 1Aa). For example, the Venn diagram for patient #1 indicates that 9 of the 891 alterations detected in the primary lesion were also detected in the cfDNA of the same patient, whereas 882 and 110 alterations were detected exclusively in primary lesion tDNA and in cfDNA, respectively. When we consider the detection rate that represents the number of genetic alterations in cfDNA divided by the number of total alterations detected in tDNA per tumor sample, the mean detectable rate in all 19 samples was 41.8% (range 1.0–69.2%). There was no correlation between the tumor genetic alteration detectability in cfDNA and tumor size (Fig. 1C). The lowermost blue circle in Fig. 1 represents the number of alterations commonly detected in both tDNA (from all tumors in that patient) and cfDNA samples from each patient (5, 24, 24, 129, 15, and 30 alterations in patients 1–6, respectively). None of these shared genetic alterations were common among all 6 patients. The following 7 genes were shared between tDNA and cfDNA of the same patient at relatively high frequencies: *ALK*, *ATM*, *ATRX*, *EGFR*, *GNAQ*, *NOTCH1*, and *ROS1*. However, the positions of the changed nucleotides and types of alteration in a specific gene were not always identical among the patients (Table 3).

**Table 3:** Genes alterations detected at high frequencies among the six patients.

Patient/gene name	*ALK*	*ATM*	*ATRX*	*EGFR*	*GNAQ*	*NOTCH1*	*ROS1*
**1**	SNP	SNP	SNP				
2					SNP	CNV	
3				SNP	SNP	CNV	CNV
4	SNP	SNP	SNP	SNP	SNP	SNP	SNP
5							
6	SNP	CNV	CNV	DEL/SNP			CNV

*SNP* single nucleotide polymorphism, *CNV* copy number variation, *DEL* deletion.

### Shared genetic alterations detected in cfDNA among the 6 patients

We attempted to elucidate the genetic alterations associated with DNA released from tumors into the bloodstream by identifying the cfDNA genetic alterations common among the 6 patients. There were 167 genetic alterations detected in cfDNA that were shared between 2 or more of the 6 patients. To focus on the biologically meaningful genetic alterations, synonymous coding single nucleotide polymorphisms and alterations located in coding, intronic, untranslated or upstream regions were excluded to confine the survey to functional alterations. In total, 15 alterations were identified (Supplementary Table 2). The alterations were frequently found in genes with tumor suppressor and DNA damage repair functions.

### The relationship between intertumoral heterogeneity harboring metastases and the ability to detect genetic alterations in cfDNA

**Figure 2:**
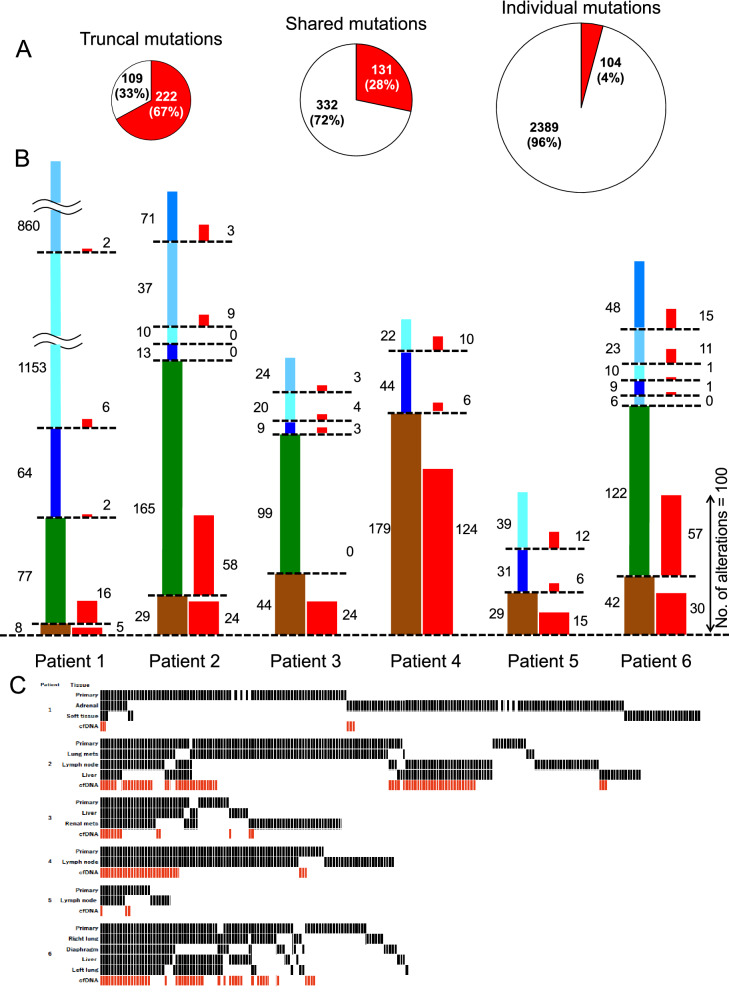
Differences in the rates of genetic alteration detection in cfDNA among truncal, shared and individual alterations. (**A**) The pie charts show the differences in the number of genetic alterations detected in all 6 patients combined according to the mutation type (truncal, shared or individual). The values represent the numbers of genetic alterations. The red colors of the charts represent the number of genetic alterations both in cfDNA and tDNA. White represents only those in tDNA. The detection rate in cfDNA was highest for individual mutations and lowest for truncal mutations. (**B**) The figures show the total number of genetic alterations in each tumor sample of each patient. The height of each column represents the proportion of genetic alterations detected, and the total column height represents the total number of genetic alterations in each patient. The brown bars represent the genetic alterations detected in all tumor lesions (truncal), green bars represent the genetic alterations detected in more than one but not all lesions (shared), and the blue bars represent the genetic alterations detected in only one lesion (individual). The red bars represent the corresponding alterations detected in cfDNA. (**C**) The heatmaps were constructed using functional alterations. One line corresponds to one genetic alteration. Abbreviations: cfDNA, cell-free DNA.

We hypothesized that intertumoral heterogeneity, which leads to differences among metastases, may influence the detection of genetic alterations in cfDNA. Genetic alterations commonly present in various tumor lesions tend to be easily detected in cfDNA because of the large number of mutant tumor cells. Some genetic abnormalities that exist only in metastatic lesions may enter the bloodstream. Thus, genetic alteration profiles in cfDNA may summarize every genetic alteration existing in every tumor of a patient, despite intertumoral heterogeneity. Genetic alterations detected in tDNA were divided into 3 groups depending on the number of tumor lesions within a single patient in whom they were detected. Mutations detected in all tumor lesions, including primary and metastatic lesions, were defined as truncal mutations. Those detected in more than 2 lesions, but not all, were defined as shared mutations. In patients whose tDNA was extracted from 2 tumor lesions (patients #4 and #5), genetic alterations from both lesions were grouped as truncal mutations. Mutations present in only 1 tumor site were defined as individual mutations. Differences in the concordance between tDNA and cfDNA in the 3 groups were analyzed using Student’s t-tests. In all 6 patients, the detection rate of genetic alterations in cfDNA was significantly higher for those also detected in tDNA. Of the mutations detected in tDNA, 67% of the truncal, 36% of the shared and 4% of the individual mutations were detected in cfDNA (Fig. 2A). The rates of tumor mutation detection in cfDNA were significantly (chi-square test with Yate’s correction) higher for truncal and shared mutations than individual mutations in all cases except patient #3 (Supplementary Table 3). In Fig. 2B, the genetic alterations detected in cfDNA that were common among the patients are shown as red columns to the right of the columns representing tDNA. Among all genetic alterations detected in tDNA, individual mutations were the most frequent (75.8% of total mutations; 96.0%, 40.3%, 27.1%, 26.9%, 70.7%, and 36.9% in patients #1–6, respectively), followed by shared mutations (14.1% of total mutations; 3.6%, 50.8%, 50.5%, and 46.9% in patients #1, 2, 3, and 6, respectively), and truncal mutations were the least common alteration (10.1% of total mutations; 0.4%, 8.9%, 22.4%, 73.1%, 29.3%, and 16.2% in patients #1–6, respectively) (Fig. 2B). Heatmaps were constructed to evaluate only biologically meaningful genetic alterations. Synonymous coding single nucleotide polymorphisms and alterations located in coding, intronic, untranslated or upstream regions were excluded to confine the data to functional alterations. Truncal mutations were also detected most frequently in cfDNA (Fig. 2C).

### The relationship between intratumoral heterogeneity in certain lesions and the ability to detect alterations in cfDNA

**Figure 3:**
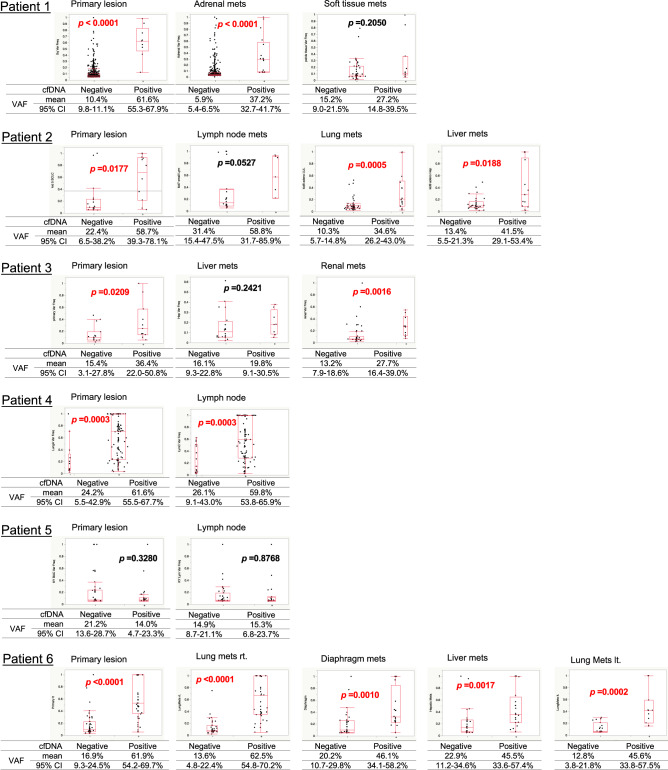
Distribution maps representing the variant allele frequencies (VAF) of each genetic alteration detected in each tumor lesion according to whether it was or was not detected in cfDNA. In 14 of the total 19 tumor lesions, the VAFs of the genetic alterations were significantly higher among those detected in cfDNA than those not detected in cfDNA. Abbreviations: cfDNA, cell-free DNA; VAF, variant allele frequency; 95% CI, 95% confidence interval; rt., right; lt., left; mets, metastasis.

The variant allele frequency (VAF) was used as a marker of intratumoral heterogeneity. VAF is calculated based on the number of copies of an allele at a specific locus. It reflects the proportion of genetic alterations, among the total alleles present, at that site in a tumor. The mean VAFs of all genetic alterations detected in tDNA in patients #1–6 were 8.6%, 24.9%, 18.5%, 57.0%, 16.8%, and 36.0%, respectively. Those detected in cfDNA were 19.0%, 20.4%, 29.1%, 50.4%, 12.2%, and 44.2%, respectively. We compared the VAF of each genetic alteration detected in tDNA between those detected in cfDNA and those not detected in cfDNA. The VAFs of tDNA alterations detected in cfDNA were significantly higher (Mann–Whitney U test) than those not detected in cfDNA in almost all tumor lesions (Fig. 3). The mean VAF of all patients’ tDNA alterations detected versus those not detected in cfDNA was 47.6% (range, 2.1–100.0) versus 4.1% (range, 1.1–100.0). In patient #1, as an example, 9 (1.0%) of the 891 genetic alterations detected in the primary lesion were also detected in cfDNA; the mean VAF of these 9 genetic alterations was 61.6% (95% confidence interval [CI], 55.3–67.9), and that of the 882 genetic alterations not detected in cfDNA was 10.4% (95% CI, 9.8–11.1; *P* < 0.0001). In the adrenal metastasis, the mean VAF of alterations detected in both the lesion and cfDNA was 37.2% (95% CI, 32.7–41.7), and that of the alterations detected in the lesion but not cfDNA was 5.9% (95% CI, 5.4–6.5; *P* < 0.0001). In the soft tissue metastasis of patient #1, the mean VAF of genetic alterations detected in both the lesion and cfDNA versus in the lesion only was 27.2% versus 15.2% (95% CI, 14.8–39.5 versus 9.0–21.5; *P* = 0.2050).

### Genetic alterations detected in cfDNA only

Some genetic alterations were detected exclusively in cfDNA, and not in the tDNA of the patient (94, 71, 36, 53, 45, and 246 alterations in patients #1–6, respectively). The VAF of the genetic alterations detected only in cfDNA was 27.7%, which was relatively low compared with that of the alterations detected in both cfDNA and tDNA: 48.2% (Supplementary Fig. 2). There were no genetic alterations detected exclusively in cfDNA that were common among all 6 patients.

### Longitudinal changes in genetic alterations detected in cfDNA

The data above showed that tumor-derived genetic alterations detected in cfDNA tended to be shared by multiple tumor lesions and to exist at a high frequency in tumor tissues. The advantage of genetic analysis using cfDNA is that it can be performed repeatedly, enabling mutation changes to be monitored over time in a patient. By confirming the longitudinal changes in genetic alteration profiles of cfDNA, it is possible to capture the changes in major genetic abnormalities, representative of the whole tumor, caused by disease progression and/or antitumor treatments.

Longitudinal changes in genetic alterations in cfDNA were investigated both at initial diagnosis and at death in 4 of the 6 patients (#1, 2, 3, and 6). The numbers of genetic alterations detected in cfDNA at diagnosis were 84, 106, 71, and 202 (patients #1, 2, 3, and 6), and these numbers increased to 119, 174, 87, and 362, respectively, just before death, that is after disease progression, in all patients. There were 17 (20.2%), 13 (12.3%), 6 (8.5%), and 184 (91.1%) genetic alterations detected at both diagnosis and just before death, indicating wide variations among the individuals. Interestingly, most of the genetic alterations detected at diagnosis were no longer apparent around the time of death. Conversely, many of the genetic alterations detected just before death were not detected at the time of diagnosis and had newly appeared during the clinical course. The numbers of such genetic alterations were 102, 161, 81, and 178 in patients #1, 2, 3, and 6, respectively. For those genetic alterations detected in the newly developed metastatic lesions but not in the primary lesion at autopsy, 3, 8, 4, and 10 alterations were already detected in cfDNA at diagnosis in patients #1, 2, 3, and 6, respectively (Table 4). These alterations were classified as shared and individual and these had a relatively high VAF in the autopsy specimens.

**Table 4:** The list of gene alterations detected both in cfDNA at diagnosis and in newly metastasized tumor (not detected in primary tumor).

Pt #	Chr	Pos_GRCh37	Ref	Variant	Gene_Name	Variant_Type	Transcript_ID	Codon_Change	AA_Change	dbSNP_ID	COSMIC_ID	snpEff_Effect	snpEff_Impact	Original organ	VAF (%)
1	chr3	41275421	T	A	*CTNNB1*	SNP	NM_001098210.1					INTRON	MODIFIER	Adrenal grand	3.1
	chr6	35420628	A	C	*FANCE*	SNP	NM_021922.2			rs9470029		INTRON	MODIFIER	Adrenal grand	31.0
														Soft tissue	12.6
	chr7	140434607	G	A	*BRAF*	SNP	NM_004333.4					INTRON	MODIFIER	Soft tissue	33.5
2	chr5	55256384	A	C	*IL6ST*	SNP	NM_002184.3	c.819T > G	p.P273			SYNONYMOUS_CODIG	LOW	Lung mets	8.7
														Liver	9.1
	chr5	56168674	G	A	*MAP3K1*	SNP	NM_005921.1					NON_SYNONYMOUS_CODNG	MODERATE	Liver	2.5
	chr5	149435535	G	T	*CSF1R*	SNP	NM_005211.3					INTRON	MODIFIER	Lung mets	22.3
														Liver	16.8
	chr5	149435536	GC	G	*CSF1R*	DEL	NM_005211.3					INTRON	MODIFIER	Lug mets	22.3
	chr7	6026775	T	C	*PMS2*	SNP	NM_000535.5	c.1621A > G	p.K541E	rs2228006		NON_SYNONYMOUS_CODIG	MODERATE	Lung mets	99.6
														Liver	99.6
	chr14	81562998	T	C	*TSHR*	SNP	NM_000369.2	c.561T > C	p.N187	rs2075179	COSM147826	SYNONYMOUS_CODNG	LOW	Lung mets	45.5
	chr14	95593158	GC	AA	*DICER1*	MNP	NM_001271282.1					INTRON	MODIFIER	Lung mets	9.2
	chr17	29559088	C	T	*NF1*	SNP	NM_001042492.2					INTRON	MODIFIER	Lung mets	49.1
														Liver	35.7
3	chr1	11181457	G	T	*MTOR*	SNP	NM_004958.3			rs17235633		INTRON	MODIFIER	Renal	54.8
	chr3	41275421	T	A	*CTNNB1*	SNP	NM_001098210.1					INTRON	MODIFIER	Renal	4.6
	chr5	149460646	T	C	*CSF1R*	SNP	NM_005211.3					INTRON	MODIFIER	Renal	6.5
	chr7	6026775	T	C	*PMS2*	SNP	NM_000535.5	c.1621A > G	p.K541E	rs2228006		NON_SYNONYMOUS_CODNG	MODERATE	Renal	99.8
6	chr2	29446231	C	T	*ALK*	SNP	NM_004304.4	c.3336G > A	p.P1112	rs146074150	COSM118189	SYNONYMOUS_CODNG	LOW	Lung mets	74.5
	chr3	187447032	G	A	*BCL6*	SNP	NM_001706.4	c.1161C > T	p.N387	rs1056932		SYNONYMOUS_CODNG	LOW	Lung mets	39.3
	chr9	98224360	C	G	*LOC100507346*	SNP	NM_038982.1			rs2274692		UPSTREAM	MODIFIER	Lung mets	69.2
	chr12	49422626	A	G	*MLL2*	SNP	NM_003482.3	c.14367T > C	p.S4789			SYNONYMOUS_CODNG	LOW	Lung mets	5.9
	chr12	49444545	G	A	*MLL2*	SNP	NM_003482.3	c.2826C > T	p.I942	rs2241726	COSM147497	SYNONYMOUS_CODNG	LOW	Lung mets	38.5
	chr12	56493822	A	C	*PA2G4*	SNP	NM_006191.2			rs2292238		UPSTREAM	MODIFIER	Lung mets	100.0
	chr16	89857964	T	C	*FANCA*	SNP	NM_000135.2			rs1800330		INTRON	MODIFIER	Lung mets	100.0
	chr16	89858024	A	G	*FANCA*	SNP	NM_000135.2			rs6500450		INTRON	MODIFIER	Lung mets	100.0
	chr19	4102449	G	A	*MAP2K2*	SNP	NM_030662.3	c.453C > T	p.D151	rs17851657	COSM1129849	SYNONYMOUS_CODNG	LOW	Lung mets	77.8
	chr22	30051657	CC	GG	*NF2*	MNP	NM_000268.3		p.GR0GG			NON_SYNONYMOUS_CODNG	MODERATE	Lung mets	69.2
														Liver	5.4

*Pt* patient, *chr* chromosome, *Ref* reference, *AA* amino acid, *SNP* single nucleotide polymorphism, *VAF* variant allele frequency, *mets* metastasis, *DEL* deletion, *MNP* manganese peroxidase.

## Discussion

Here, we examined 6 patients with advanced NSCLC who harbored EGFR mutations. We evaluated the correlations between the genetic alteration profiles detected in cfDNA and those detected in whole tumor lesions, including distant metastases obtained from autopsy samples. The results yielded meaningful insights into the factors that regulate the detection sensitivity of cfDNA-based genetic testing in patients with advanced NSCLC. Using autopsy specimens, we obtained comprehensive genetic alteration profiles, even in distant metastases, in individual patients, regardless of the presence of tumoral heterogeneity. Some studies have demonstrated intra- or intertumoral heterogeneity in malignant cancers^11–14^. Such heterogeneity can cause malignant behavior and resistance to antitumor agents. The tumor-derived genetic alterations used as a control for mutation analyses in cfDNA reflect only a small portion of the overall alterations in all tumors of a patient with advanced NSCLC. This result is attributable to the fact that tumor specimens collected in clinical practice via transbronchial and CT-guided biopsies represent a very small portion of the tumor. Therefore, genetic testing based on tissue biopsies may not be accurate owing to intra- or intertumoral heterogeneity; as a result, important genetic alterations may be missed. If the entire suite of tumor-derived genetic alterations, including those in both primary and metastatic lesions, are present in cfDNA, then the latter offers a useful method for more accurate evaluation of genetic alterations in individual patients. In a phase III clinical trial analyzing recurrent EGFR mutant lung cancer, specificity and sensitivity of molecular ctDNA testing using NGS for detection *EGFR* sensitizing alterations were 99% (for L858R) and 99% (for exon 19 deletion), respectively, and 62% (for L858R) and 81% (for exon 19 deletion), respecitvely^15^. Concordance was reported to be 79–80%^16,17^. Furthermore, investigation tens of cancer-related genes using molecular ctDNA testing by NGS from patients with NSCLC showed a specificity of 87–99% and sensitivity of 58–85%^18,19^. These results were similar or superior to other methods, including COBAS (Roche Molecular Diagnostics, Branchburg, NJ, USA), digital droplet polymerase chain reaction (PCR), quantitative PCR, and beads emulsions amplification and magnetics^20^. Several reports have discussed the implications of tumor heterogeneity^10,11^. However, more thorough analyses of tumor genetic alterations are achievable with ctDNA.

This study revealed 2 innovative findings in regard to the features of genetic alterations detectable in cfDNA. First, the detection rate of alterations in cfDNA was highest for truncal and lowest for individual mutations. This indicates that genetic alterations present in multiple lesions including metastases are more likely to be released into the bloodstream and to be detected by cfDNA-based genetic testing. Second, the intratumoral VAF of the genetic alterations detected in cfDNA was clearly higher than that of the alterations not detected in cfDNA. Genetic alterations homogeneously present in tumor tissue have a higher chance of being detected in cfDNA, considering that they are more abundant and thus likely to enter the bloodstream. Using tDNA from multiple lesions and serial cfDNA samples from a case of metastatic breast cancer, Murtaza et al. showed that stem mutations were more likely to be detected than private mutations by analyzing one breast cancer patient’s ctDNA using NGS^11^. The broad prevalence and high VAFs of genetic alterations may not be an independent feature of advanced cancer because both of these factors are meaningful and central for overall tumor evolution. In another analysis of 4 patients with NSCLC, the variants existing simultaneously among multiple tumor lesions including distant metastases tended to have a higher VAF in tDNA than that of the variants detected in only one organ (3.85% versus 0.88%)^21^. The genetic alterations detected in cfDNA in all patients included those in EGFR, which is a typical driver oncogene found in NSCLC. The presence of activating EGFR mutations in the tumor tissues, which were readily detected in cfDNA, is consistent with the 2 features described above. We speculate that genetic alterations, such as activating EGFR mutations, that are fundamental to and characteristic of tumorigenesis are widely present in tumor tissues and may also be detected in cfDNA. The genetic alterations with a higher VAF in tDNA may play an important role during early carcinogenesis. We clearly showed that the higher the VAF of individual genetic alterations in tumor tissues, the easier those alterations are to detect in cfDNA. Moreover, tumor samples collected at autopsy were more reliable than clinical biopsy specimens. The mean VAF of tDNA genetic alterations that were also detectable in cfDNA was 47.6%, indicating that approximately half of the tumor cells did not carry that genetic alteration. This result also demonstrated the limitation of genetic testing using small and tiny tumor biopsies in terms of intratumoral heterogeneity. Thus, gene mutation profiles in cfDNA, compared with tumor tissues, provide more varied genetic alterations, enhancing our understanding of tumor progression. We predict that tumor-derived genetic alterations present in multiple tumors (i.e., truncal mutations) and with a high VAF in tumor tissues may be important characteristics of a specific tumor, and the genetic alterations of that tumor may be enriched in cfDNA, which is potentially an important biospecimen for identifying biomarkers and an alternative sample to tumor tissue.

Similar results have already been reported^10,11,22,23^; however, we believe that our results provide a clearer indication of the characteristics of ctDNA. Previous data have shown that the genetic abnormalities detected in cfDNA are easily detected as truncal mutations and that such abnormalities are readily detected as genetic alterations with high intratumoral VAFs^11,24^. However, these reports used biopsy tissue, and analyses were thus performed using only a portion of the tumor tissue. In addition, many of these studies focused on the fact that cfDNA could be a surrogate tissue for tumor tissue. From the outset, our aim has been to determine the characteristics of genetic alterations in cfDNA. By using 6 autopsy cases, these findings are made clearer by using multiple tumor tissues obtained at the same time and by adding statistical analysis to the results obtained from the 6 cases. In this way, we were able to show these findings more clearly.

Based on the analyses of cfDNA, 15 genetic alterations were commonly detected in cfDNA from all 6 patients (Supplementary Table 2). These genetic alterations are tumor suppressor genes or related to DNA repair functions. cfDNA is useful for monitoring the presence of tumor-derived genetic alterations, plus tumor-derived fragments of DNA may possess a biological role. Garcia-Olmo et al. showed that NIH-3T3 cells, derived from mouse embryonic fibroblasts, internalized nucleic acids harboring a mutated KRAS gene when placed in contact with plasma samples from patients with KRAS-mutant colorectal cancers in vitro^25^. Those authors proposed that tumor-derived cfDNA fragments may be directly involved in cancer metastasis. Similarly, exosomes, which are small membrane vesicles originating from large multivesicular bodies, have attracted attention as a promising biomarker. One of the molecular mechanisms of tumor metastasis is the transfer of oncogenic growth factor receptors or their ligands^26^.

One of the objectives of this study was to determine whether cfDNA genetic testing is capable of summarizing all gene alterations within an individual patient despite the presence of tumoral heterogeneity. The rates of shared (36%) and individual mutations (4%) detected in this study are by no means low, and these genetic abnormalities could be missed in a single tumor biopsy sample (Fig. 2). Thus, cfDNA genetic testing detects part of the intertumoral heterogeneity within an individual patient. In a study of the KRAS mutation status in 12 cases of metastatic colorectal cancer, the concordance of mutations detected between cfDNA and the primary tumor and between cfDNA and the metastases were 39% and 55%, respectively^27^. Similarly, our study showed that not all alterations detected in tDNA were detected in cfDNA.

In our longitudinal analysis comparing the cfDNA mutation profiles at diagnosis with those at death, the number of alterations that disappeared or were newly acquired during the clinical course was higher than that of those detected both at diagnosis and death. Thus, the gene mutation profiles in cfDNA showed dynamic longitudinal changes during the clinical course. EGFR mutations were detected both at the time of diagnosis and just before death in every patient. The driver and fundamental genetic alterations may be stable and thus readily detected in cfDNA throughout the treatment course. Moreover, some genetic alterations (3, 8, 4, and 10 alterations in patient #1, 2, 3, and 6, respectively) that were detected only in metastatic lesions occurring after resistance development were detected in cfDNA; these alterations were not detected in the primary tumor tissue at the time of diagnosis (Table 4) and tended to have high VAFs in the distant metastases (Table 4). The alterations detected in *MAP3K1*, *PMS2*, and *NF2* were non-synonymous coding mutations, and these may play a role in promoting distant metastasis. MAP3K1 regulation of cell motility is mediated by the protease calpain localized at focal adhesions. Loss of MAP3K1 expression reduces cell migration and invasion and delays tumor metastasis^28^. The *PMS2* gene is a member of a set of genes known as mismatch repair genes and encodes a protein that plays an essential role in repairing DNA^29^. The *NF2* gene encodes the merlin protein, which suppresses cell proliferation and promotes cell adhesion^30^. These mutations were not detected in primary tDNA at diagnosis, perhaps because they were not included in the tumor tissue biopsy specimens collected at diagnosis. The tumor cells harboring these mutations may have existed as minor clones in tumor tissues at the time of diagnosis. Subsequently, these mutations may have promoted distant metastasis of these tumor cells, which eventually developed into major clones and released the mutated DNA as cfDNA. Alternatively, a small metastatic lesion may have been present at diagnosis but went undetected at that time, and the mutated DNA specific to the metastatic lesion was released as cfDNA. This metastatic lesion then grew over the course of treatment and was detected at autopsy. Although a few genetic alterations that emerged in the newly developed metastatic tumor lesions were already detected in cfDNA at initial diagnosis, as shown in Table 4, there was no attempt to extract these rare alterations among those from cfDNA. Tracing the specific alterations corresponding to new metastatic lesions is difficult. Thus, this represents a limitation to our study, and further work is needed to fully elucidate cancer evolution using longitudinal cfDNA analyses. Monitoring genetic alterations in cfDNA is expected to reveal the “important” evolving properties of a tumor, such as progression of distant metastases or resistance to antitumor agents, at the time of sampling.

Although we obtained meaningful results from the cfDNA genetic analyses, many problems must be solved in liquid biopsy. To be used in general clinical practice, cfDNA-based liquid biopsy must be established as a reliable tool for detection of early disease as well as metastases and for prediction of prognosis. The establishment of a method for identifying metastatic sites using cfDNA will provide more useful and innovative clinical information, such as which tumor lesions are shrinking or developing drug resistance. Furthermore, if we can identify the organ from which a ctDNA originated, we can obtain more useful information for detailed assessments of tumors, including metastases, during the treatment course by blood sampling. Snyder et al. focused on nucleosome occupancy in cfDNA and its correlations with nuclear architecture, gene structure and expression in cells, suggesting the cell type of origin^31^. Further investigations are needed to develop a technique to distinguish genetic alterations in ctDNA among individual metastases.

cfDNA-based EGFR mutation tests have been used in advanced NSCLC to establish a prognosis and assess resistance to anti-cancer agents. The mechanisms of acquired resistance to first- and second-generation EGFR-TKIs are well understood. The most common mechanism is development of a secondary T790M mutation, which is found in half of the patients with EGFR-TKI resistance. Other mechanisms include small cell carcinoma transformation, gene amplification of *EGFR*, *ERBB2*, and *MET* and additional mutations in *PI3K*, *BRAF*, and *KRAS*^32–36^. cfDNA-based testing of the EGFR T790M mutation has already been approved for determining treatment for NSCLC^15,37–43^. Moreover, cfDNA-based tests of other resistance mechanisms, such as resistance-associated gene amplification and small cell transformation, are under investigation. *HER2* amplification in metastatic breast and colorectal cancers can be detected by digital PCR analysis of plasma ctDNA^44,45^. For detection of transformed small-cell carcinoma, techniques for circulating tumor cell analysis may be needed to investigate the expression levels of proteins or mRNAs. A validation study of a method to identify resistance mechanisms other than T790M mutation development will be needed in the near future.

One of the limitations of this study was the small sample size (6 patients), which was insufficient to elucidate the characteristics of cfDNA in cancer patients. Paired samples of tissue specimens from multiple tumor lesions and cfDNA collected from patients at autopsy may be used to evaluate cfDNA characteristics and perform gene analyses, and sufficient DNA for gene analyses can be collected from autopsy samples. Furthermore, it is difficult to enroll a large number of postmortem patients whose blood samples for this study were collected before death. The other limitation is that the patients in this study had strong oncogenic driver mutations in EGFR. The results of our study might be biased and may not reflect the genomic characteristics of all lung cancer cases, such as those harboring KRAS mutations, a major genetic alteration in lung adenocarcinoma and smoking-related lung cancer. Thus, it remains unclear whether our results apply to patients with cancers originating from organs other than the lung. As a final limitation, it should be noted that only a portion of the metastases were sampled because at the time of death they were too numerous for exhaustive sampling.

In conclusion, this study evaluated mutations in cfDNA and compared them with tDNA obtained from autopsy samples. The investigation revealed that tumor-derived genetic alterations most easily detected in cfDNA were truncal mutations with a high VAF. Liquid biopsy of cfDNA for genetic testing in cancer patients has the potential advantage of detecting important genetic abnormalities enriched in cfDNA that are characteristic of the cancer cells present in a patient.

## Methods

### Patient selection

The patients enrolled in this study were selected according to the following criteria: (1) autopsy cases with histologically confirmed metastatic primary lung cancer, (2) death caused by cancer progression, (3) cfDNA extracted from stored plasma samples collected within the 3-month period before death, and (4) written informed consent obtained from the patient during her or his lifetime of the protocol approved by the institutional review board of Kanazawa University Graduate School of Medicine (approval # 327). The protocol for the NGS-based genotyping assays used in tumor tissues and cfDNA from patients with advanced NSCLC applied to specimens obtained at autopsy. We collected the following clinical data: sex, smoking history, diagnosis date, histological type, treatment regimen for lung cancer and ages at diagnosis and death. The study was conducted in accordance with the provisions of the Declaration of Helsinki.

### Tumor tissue collection and DNA extraction

All tissue samples obtained at autopsy were diagnosed histologically by a pathology expert. The tumor size, which was represented by the maximal diameter of the tumor, was measured in CT images that were scanned within ten days of death. DNA was extracted from frozen tumor samples using a QIAAMP DNA MINI KIT (Qiagen) and from formalin-fixed paraffin-embedded (FFPE) tumor samples using the QIAAMP DNA FFPE TUMOR TISSUE KIT (Qiagen) according to the manufacturer’s instructions. The extracted DNA was stored at − 20 °C until used. The concentration and quality of the DNA were measured using a QUBIT 2.0 FLUOROMETER (Life Technologies) with DSDNA HS ASSAY KITS (Life Technologies) and QIASEQ DNA QUANTIMIZE ARRAY KIT (Qiagen).

### Blood collection and extraction of cfDNA and germline DNA

Blood samples were collected within 3 months of death from all enrolled patients, and cfDNA was extracted at initial diagnosis prior to systemic antitumor-treatment (including EGFR-TKIs). Whole blood (12 mL) was collected in ethylenediaminetetraacetic acid tubes and centrifuged at 700 × *g* (2000 rpm) for 10 min within 2 h of blood collection, as described previously^37^. cfDNA was extracted immediately from the plasma supernatant (4 mL) using a QIAAMP CIRCULATING NUCLEIC ACID KIT (Qiagen), and germline DNA was extracted from whole blood cells, obtained as the pellet in centrifugation, using a QIAAMP DNA MINI KIT (Qiagen). The extracted DNA was stored at − 20 °C until use. The concentration and quality of cfDNA and germline DNA were measured using the abovementioned methods.

### Detection of genetic alterations

Tumor genotyping assays by NGS were performed using a GENEREAD DNASEQ TARGETED PANELS V2 HUMAN COMPREHENSIVE CANCER PANEL (Qiagen). According to the manufacturer’s instructions, each sample used for sequencing, including cfDNA, contained 40 ng DNA. The PCR products and constructed libraries were analyzed for size range and molarity using an AGILENT 2100 BIOANALYZER. Sequencing was performed using MISEQ with a MISEQ REAGENT KIT V2 (300-CYCLES) for paired-end 151-base pair and HISEQ 2000 with paired-end 100-base pair platforms (Illumina). The data were analyzed using GENEREAD DNASEQ ANALYSIS SOFTWARE (Qiagen) for read mapping and post-processing, variant calling and filtering, and annotation. For variant calling and filtering, the GATK Unified Genotyper program (GATKLite version 2.3-9) was used for calling variants on individual samples, and Strelka^46^ was used with default parameters to call somatic variants from matched tumor and normal samples. All of the genetic alterations detected in each sample were reported with high, medium or low confidence, according to Qiagen’s GENEREAD DNASEQ ANALYSIS SOFTWARE (Qiagen). The detected gene aberrations were distinguished per nucleotide changes, not only per genes. Further analysis was performed only on high confidence genetic alterations. We identified somatic alterations in tumor tissues to exclude germline alterations, derived from the patient’s germline DNA, observed in crude alterations prepared from each tumor. To compare the genetic alterations between tumor samples, we used an original script in “Terminal app”, a terminal emulator in the MacOS operating system (Supplementary File 32). The terminal command dictated coordination of the same genetic alteration between samples.

Within individual patients, the concordance and discordance of each gene mutation between tDNA and cfDNA was analyzed, and genetic alterations shared among all tumor tissues (primary lesions and all metastatic lesions) and cfDNA samples were selected. Then, the genetic alterations were divided into 3 groups according to the detection frequency in tumor samples from a single patient. Mutations present in all tumor sites including the primary lesion and metastases were defined as truncal. Those present in more than 2 sites but not all sites were defined as shared branch mutations. Those present at only a single site were defined as individual branch mutations. Differences in the concordance between tDNA and cfDNA in the 3 groups were analyzed by Student’s t-test. Correlations between the VAF of a mutation and its detection in cfDNA were analyzed by Student’s t-test. The VAF was calculated based on the allele coverage as follows: VAF = (allele coverage of the variant call)/(sum of the allele coverage of the reference and that of the variant call). We used the Mann–Whitney U test to compare the VAF of a genetic alteration detected in both tDNA and cfDNA with that of an alteration detected in tDNA only (and not cfDNA).

### Statistical analysis

All statistical analyses were conducted using JMP 12. A value of *P* < 0.05 was considered statistically significant.

## Supplementary Information

Supplementary Files.

Supplementary Figures and Tables.

## Data Availability

All of the data that support the findings of this study are available.
