# Supplementary material for: Molecular features of tumor-derived genetic alterations in circulating cell-free DNA in virtue of autopsy analysis
Source: Sci Rep. 2021 Apr 16;11:8398. doi: 10.1038/s41598-021-87094-1 (PMC8052353; doi:10.1038/s41598-021-87094-1)
Supplement: Supplementary file 2 — Supplementary Figures and Tables. [file 41598_2021_87094_MOESM2_ESM.pdf]

Title

Molecular features of tumor-derived genetic alterations in circulating cell-free DNA in virtue of autopsy analysis

Author list

Hayato Koba

Hideharu Kimura

Taro Yoneda

Takashi Sone

Noriyuki Ohkura

Johsuke Hara

Kazuyoshi Hosomichi

Atsushi Tajima

Kazuo Kasahara

Supplementary Fig. 1. Bioanalyzer image of library.

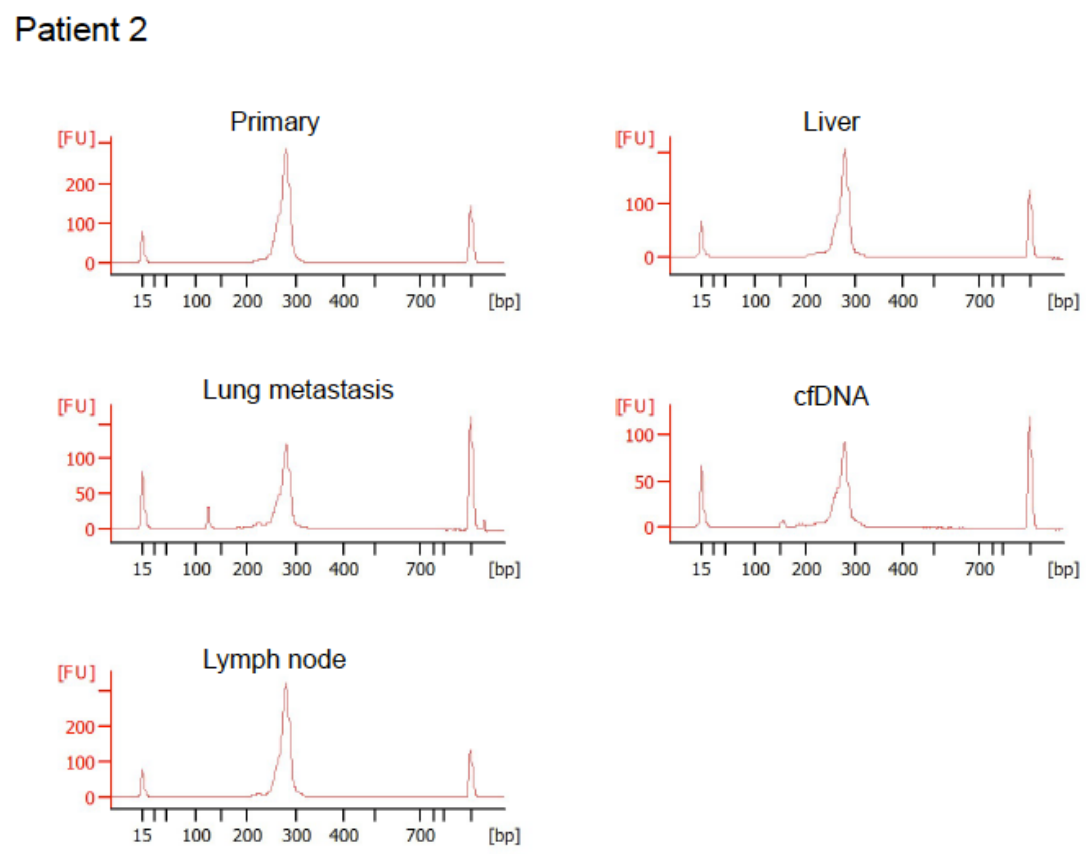

Bioanalyzer image of library. Examples of library quality from the NGS analysis conducted in patient #2 using the BIOANALYZER HIGH SENSITIVITY DNA ASSAY KIT (Agilent Technologies). Each library exhibited a single peak around 280 bp and had a sufficient concentration to perform NGS analysis.

Supplementary Fig 2. Distribution maps showing the variant allele frequencies (VAFs) of the genetic alterations in cfDNA.

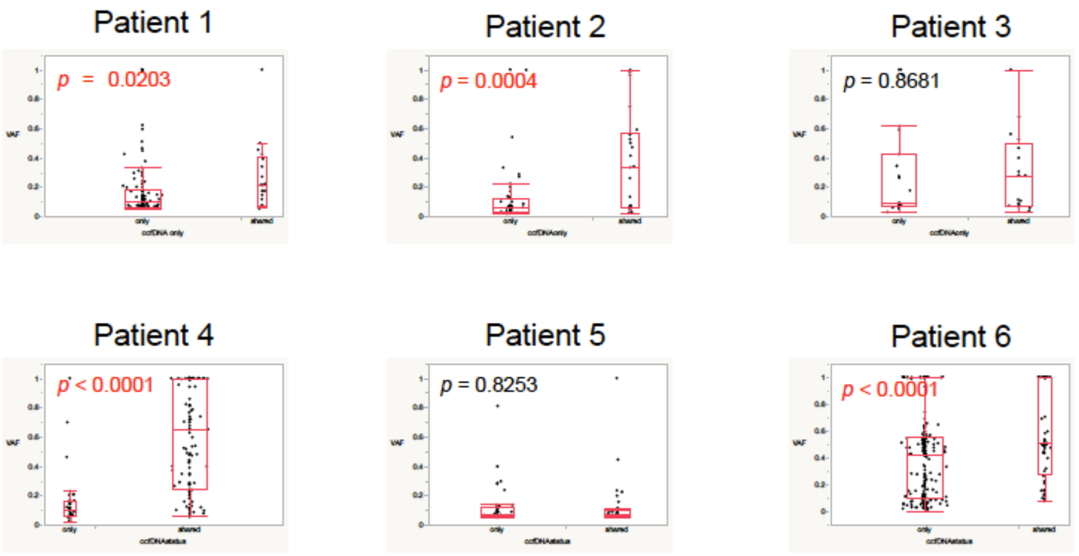

Distribution maps showing the variant allele frequencies (VAFs) of the genetic alterations in cfDNA according to whether the alterations were detected exclusively in cfDNA (left box plot) or in both cfDNA and tDNA (right box plot). In four patients (#1, 2, 4 and 6), the VAFs of the alterations detected exclusively in cfDNA were significantly lower than those of the alterations detected in both cfDNA and tDNA. The Mann–Whitney *U* test was used to compare the VAF of a genetic alteration detected in both tDNA and cfDNA with that of an alteration detected in cfDNA only (and not tDNA).

Supplementary Table 1. List of the genes included in the GENEREAD DNASEQ TARGETED PANELS V2 HUMAN COMPREHENSIVE CANCER PANEL (QIAGEN).

ABL1	*BUB1B*	*DDR2*	*FGFR2*	*IDH2*	*MEN1*	*PDGFRA*	*SMARCA4*
AKT1	*CARD11*	*DICER1*	*FGFR3*	*IKZF1*	*MET*	*PHF6*	*SMARCB1*
AKT2	*CBL*	*DNMT3A*	*FH*	*IL6ST*	*MLH1*	*PIK3CA*	*SMO*
ALK	*CBLB*	*ECT2L*	*FLCN*	*IL7R*	*MSH2*	*PIK3R1*	*SPOP*
AMER1	*CD79A*	*EGFR*	*FLT3*	*JAK1*	*MSH6*	*PMS2*	*SRC*
APC	*CD79B*	*EP300*	*FUBP1*	*JAK2*	*MTOR*	*PPP2R1A*	*STK11*
AR	*CDC73*	*EPCAM*	*GATA1*	*JAK3*	*MUTYH*	*PRDM1*	*SUFU*
ARID1A	*CDH1*	*ERBB2*	*GATA2*	*KDM6A*	*MYC*	*PRKAR1A*	*TERT*
ARID2	*CDK12*	*ERBB3*	*GATA3*	*KDR*	*MYD88*	*PTCH1*	*TNFAIP3*
ASXL1	*CDK4*	*ERBB4*	*GNA11*	*KIT*	*NF1*	*PTEN*	*TNFRSF14*
ATM	*CDKN2A*	*ERCC5*	*GNAQ*	*KLF6*	*NF2*	*PTPN11*	*TP53*
ATRX	*CHEK2*	*ESR1*	*GNAS*	*KMT2D*	*NFE2L2*	*PAC1*	*TSC1*
BAP1	*CIC*	*EZH2*	*GPC3*	*KRAS*	*NFKBIA*	*RB1*	*TSC2*
BCL6	*CREBBP*	*FAM46C*	*GRIN2A*	*MAP2K1*	*NOTCH1*	*RET*	*TSHR*
BCOR	*CRLF2*	*FANCA*	*H3F3A*	*MAP2K2*	*NOTCH2*	*ROS1*	*U2AF1*
BRAF	*CSF1R*	*FANCD2*	*HIST1H3B*	*MAP2K4*	*NPM1*	*SDHB*	*VHL*
BRCA1	*CTNNB1*	*FANCE*	*HNF1A*	*MAP3K1*	*NRAS*	*SETD2*	*WT1*
BRCA2	*CYLD*	*FAS*	*HRAS*	*MAP4K3*	*PALB2*	*SF3B1*	*XPC*
BRIP1	*DAXX*	*FBXO11*	*HSPH1*	*MDM2*	*PAX5*	*SLC7A8*	*ZNF2*
BTK	*DDB2*	*FBXW7*	*IDH1*	*MED12*	*PBRM1*	*SMAD4*	*ZRSR2*

Supplementary Table 2. Genetic alterations shared between the cfDNA samples of at least two patients

Gene_Name	Variant_Type	AA_Change	COSMIC_ID	snpEff_Effect	function
ALK	SNP	p.I1461V		NON_SYNONYMOUS_CODING	Oncogene, Immune response, RTK
ZNF2	DEL	p.LR156L		CODON_DELETION	Transcription factor
APC	SNP	p.V1822D		NON_SYNONYMOUS_CODING	Tumor Suppressor gene, Adhesion, Apoptosis, Cell cycle, DNA damage & repair
EGFR	SNP	p.R521K		NON_SYNONYMOUS_CODING	Oncogene, Adhesion, RTK
ATM	SNP	p.N1983S		NON_SYNONYMOUS_CODING	Tumor Suppressor gene, DNA damage & repair
BRCA2	SNP	p.V2466A		NON_SYNONYMOUS_CODING	Tumor Suppressor gene, DNA damage & repair
BIVM-ERCC5	SNP	p.G1507R		NON_SYNONYMOUS_CODING	Nucletide excision repair
BIVM-ERCC5	SNP	p.G1534R		NON_SYNONYMOUS_CODING	
TSHR	SNP	p.E727D		NON_SYNONYMOUS_CODING	Oncogene, G-protein coupled receptor signaling
FANCA	SNP	p.G809D	COSM435949	NON_SYNONYMOUS_CODING	DNA damage & repair
FANCA	SNP	p.G501S		NON_SYNONYMOUS_CODING	
FANCA	SNP	p.T266A		NON_SYNONYMOUS_CODING	
TP53	SNP	p.P72R	COSM250061	NON_SYNONYMOUS_CODING	Tumor Suppressor gene, Cell cycle, DNA damage & repair, Immune response, Transcription factor
BRIP1	SNP	p.S919P		NON_SYNONYMOUS_CODING	DNA damage & repair
ASXL1	SNP	p.L815P		NON_SYNONYMOUS_CODING	Tumor Suppressor gene, Epigenetics

Abbreviations: AA, amino acid; SNP, single nucleotide polymorphism; RTK, receptor tyrosine kinases; DEL, deletion; MNP, manganese peroxidase.

Supplementary Table 3. The differences in mutation detection rates in cfDNA between common mutations (truncal or shared) and individual mutations in each patient.

Patient 1	cfDNA positive	cfDNA negative	
Truncal or shared	21	64	P < 0.0001
Individual	10	2067	

Patient 2	cfDNA positive	cfDNA negative	
Truncal or shared	82	112	P < 0.0001
Individual	21	110	

Patient 3	cfDNA positive	cfDNA negative	
Truncal or shared	24	119	P = 0.8966
Individual	10	43	

Patient 4	cfDNA positive	cfDNA negative	
Truncal or shared	124	55	P < 0.0001
Individual	16	50	

Patient 5	cfDNA positive	cfDNA negative	
Truncal or shared	15	14	P = 0.0236
Individual	18	52	

Patient 6	cfDNA positive	cfDNA negative	
Truncal or shared	87	77	P = 0.0006
Individual	29	67	

P values were calculated by the chi-square test with Yate’s correction.

Supplementary Files.

File 1-31 are all genetic alteration profile from all samples. File number corresponds the following:

1. Primary lesion of patient #1
2. Adrenal metastasis of patient #1
3. Soft tissue metastasis of patient #1
4. cfDNA of patient #1
5. Germline DNA of patient #1
6. Primary lesion of patient #2
7. Lung metastasis of patient #2
8. Lymph node metastasis of patient #2
9. Liver metastasis of patient #2
10. cfDNA of patient #2
11. Germline DNA of patient #2
12. Primary lesion of patient #3
13. Liver metastasis of patient #3
14. Renal metastasis of patient #3
15. cfDNA of patient #3
16. Germline DNA of patient #3
17. Primary lesion of patient #4
18. Lymph node metastasis of patient #4
19. cfDNA of patient #4
20. Germline DNA of patient #4
21. Primary lesion of patient #5
22. Lymph node metastasis of patient #5
23. cfDNA of patient #5
24. Germline DNA of patient #5
25. Primary lesion of patient #6
26. Right lung metastasis of patient #6
27. Left lung metastasis of patient #6
28. Diaphragm metastasis of patient #6
29. Liver metastasis of patient #6
30. cfDNA of patient #6
31. Germline DNA of patient #6.

File 32 is an original “terminal app” script.
